# Safety and pharmacokinetics of SPR206 in subjects with varying degrees of renal impairment

**DOI:** 10.1128/aac.00505-23

**Published:** 2023-10-12

**Authors:** Jon B. Bruss, Justin Bader, Kamal A. Hamed

**Affiliations:** 1 Spero Therapeutics, Inc., Cambridge, Massachusetts, USA; University of Pittsburgh, Pittsburgh, Pennsylvania, USA

**Keywords:** polymyxins, SPR206, pharmacokinetics, safety, tolerability

## Abstract

SPR206 is a novel polymyxin derivative with potent *in vitro* activity against susceptible and multidrug-resistant strains of *Acinetobacter baumannii*, *Pseudomonas aeruginosa*, *Klebsiella pneumoniae*, *Escherichia coli*, and *Enterobacter* species. SPR206 is eliminated renally. The safety, tolerability, and pharmacokinetics (PK) of SPR206 were evaluated in healthy subjects with normal renal function (Cohort 1) and subjects with varying degrees of renal impairment (RI) (Cohorts 2–4) or end-stage renal disease (ESRD) on hemodialysis (HD) (Cohort 5). Subjects in Cohorts 1–4 received a 100-mg intravenous (IV) dose of SPR206. Subjects in Cohort 5 received a 100-mg IV dose within 2 h after HD on day 1 and 1 h before HD on day 5. Safety and PK analyses included 37 subjects. Mostly mild but no serious treatment-related adverse events were reported. Systemic exposure to SPR206 increased as renal function decreased, with mean area under the concentration-time curve from time 0 to the last quantifiable concentration (AUC_0–last_) values 39% to 239% greater in subjects with RI vs healthy subjects. Mean plasma clearance (CL) of SPR206 decreased with decreasing renal function (29% to 76% lower vs healthy subjects). In subjects with ESRD, AUC_0–last_ decreased by 51%, and CL increased by 92% for dialyzed vs nondialyzed conditions. SPR206 was excreted in urine within 12 h in healthy subjects and subjects with mild RI (Cohort 2) but was prolonged in those with moderate and severe RI (Cohorts 3 and 4, respectively). In summary, SPR206 was generally safe and well tolerated, and the PK of SPR206 was well characterized in subjects with RI.

## INTRODUCTION

Antimicrobial resistance is a growing problem worldwide, and carbapenem-resistant *Acinetobacter baumannii*, multidrug-resistant (MDR) *Pseudomonas aeruginosa*, and carbapenem-resistant and extended-spectrum beta-lactamase-producing Enterobacterales have been identified as urgent or serious threats by the Centers for Disease Control and Prevention and as critical priority by the World Health Organization (1, 2). Bacteremia, hospital-acquired and ventilator-associated bacterial pneumonia (HABP/VABP), and complicated urinary tract infections are often caused by MDR *A. baumannii* (3 – 7) and account for excess morbidity and mortality (7 – 14). Similarly, *P. aeruginosa* causes serious infections with increased rates of morbidity and mortality (15, 16). Thus, an urgent need exists to identify new and safer antimicrobial agents to treat serious infections due to these MDR pathogens (1, 2, 17 – 23).

With the continuing emergence and spread of MDR and specifically carbapenemase-producing clinical isolates of Enterobacterales, *P. aeruginosa*, and *A. baumannii*, additional treatment options are needed. At present, most carbapenem-resistant strains of *Klebsiella pneumoniae*, *Escherichia coli*, *P aeruginosa*, and *A. baumannii* are still susceptible to the available polymyxin antibiotics (polymyxin B and colistin), and consequently, these agents continue to be utilized in the treatment of serious Gram-negative infections (24, 25). However, toxicity, particularly nephrotoxicity, restricts their use, and polymyxins are often reserved as a last line of defense therapy (26).

SPR206 is a novel polymyxin derivative with activity against many Gram-negative pathogens, including susceptible and MDR strains of *A. baumannii*, *P. aeruginosa*, *K.  pneumoniae*, *E. coli*, and *Enterobacter* species that pose an increased risk for antimicrobial resistance (27 – 32). *In vitro* and *in vivo* pharmacology studies suggest that SPR206 has more potent activity than colistin and has at least similar or more potent activity than polymyxin B (33 – 37). The MIC_90_ values for SPR206 vs colistin against *Acinetobacter* species, *P. aeruginosa*, and Enterobacterales clinical isolates were 0.5 vs 1, 0.25 vs 1, and 0.25 vs 0.5 mg/L, respectively (36, 37). In murine lung and thigh infection models caused by MDR *A. baumannii* and *P. aeruginosa* and ascending urinary tract infection model caused by *E. coli*, SPR206 showed similar or superior bacterial burden reductions than polymyxin B (33, 34). Additionally, nonclinical studies suggest the potential for an improved safety profile with SPR206 compared to polymyxin B (38, 39). In nonclinical studies, SPR206 was generally safe and well tolerated at exposures required for efficacy, with a low risk for respiratory, central nervous system, and cardiovascular events, and exhibited a low risk for clinically significant drug-drug interactions. In a Phase 1 study, SPR206 was well tolerated and revealed a dose-proportional pharmacokinetic (PK) profile (40). There was no evidence of changes in renal function or renal impairment (RI) with intravenous (IV) single doses up to 400 mg or multiple doses of 100 mg IV every 8 h for up to 14 days. SPR206 is being developed as an IV formulation for the treatment of serious infections of the respiratory tract, namely, HABP/VABP.

The primary objective of this study was to evaluate the safety, tolerability, and PK of SPR206 100-mg single IV dose in subjects with normal renal function (Cohort 1), subjects with varying degrees of RI (Cohorts 2–4), and subjects with end-stage renal disease (ESRD) on hemodialysis (HD) (Cohort 5).

## RESULTS

Thirty-seven subjects were enrolled and included in the safety and PK populations. Baseline characteristics were generally similar across cohorts except for renal function, with 16 (43.2%) females, mean [standard deviation (SD)] age of 57.5 (12.2) years, and mean (SD) body mass index (BMI) of 28.2 (5.0) kg/m^2^ (Table 1). In Cohort 5, the mean duration of the HD session was 251 min (range, 240–275 min; median, 240 min).

**TABLE 1:** Baseline characteristics

	Cohort 1, normal renal function, eGFR^*a*^ ≥90 mL/min/1.73 m^2^(*n* = 8)	Cohort 2,mild renalimpairment, eGFR^*a*^ 60–<90 mL/min/1.73 m^2^ (*n* = 8)	Cohort 3, moderate renal impairment, eGFR^*a*^ 30–<60 mL/min/1.73 m^2^ (*n* = 6)	Cohort 4, severe renal impairment, eGFR^*a*^<30 mL/min/1.73 m^2^ (*n* = 8)	Cohort 5, end-stage renal disease on hemodialysis(*n* = 7)
Age, years^*b*^	52.6 ± 9.8	51.3 ± 12.7	64.5 ± 8.3	65.3 ± 14.6	57.5 ± 12.2
Age range, years	42–69	34–74	51–75	48–75	35–76
Female, n (%)	2 (25.0)	6 (75.0)	2 (33.3)	2 (25.0)	4 (57.1)
Body weight, kg^*b*^	79.3 ± 8.7	67.7 ± 11.3	76.5 ± 19.4	80.9 ± 9.1	91.5 ± 21.4
Body mass index, kg/m^2*b*^	27.0 ± 2.7	24.8 ± 3.8	27.6 ± 5.4	29.3 ± 4.6	32.5 ± 6.0
Race, n (%)					
White	5 (62.5)	8 (100)	6 (100)	8 (100)	4 (57.1)
Native Hawaiian or other Pacific Islander	0	0	0	0	1 (14.3)
Other	3 (37.5)	0	0	0	2 (28.6)
Hispanic or Latino, n (%)	1 (12.5)	2 (25.0)	6 (100)	8 (100)	7 (100)
eGFR^*a*^, mL/min/1.73 m^2*b*^	100.6 ± 4.3	72.1 ± 7.2	47.2 ± 6.9	19.8 ± 6.1	Not applicable
Creatinine clearance, mL/min^*b*^	116.6 ± 18.1	80.0 ± 4.4	61.4 ± 16.6	25.7 ± 6.7	12.8 ± 2.6

^*a*^ eGFR, estimated glomerular filtration rate.

^*b*^ Mean ± standard deviation.

### Safety/tolerability

**Fig 1:**
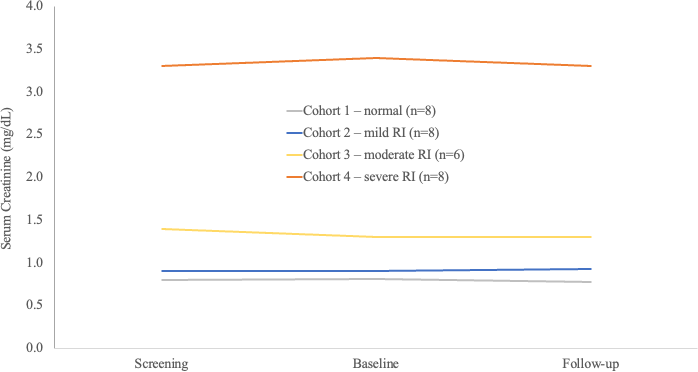
Mean serum creatinine concentrations at screening, baseline, and follow-up visits for Cohorts 1–4. RI, renal impairment.

At least one treatment-emergent adverse event (AE) (TEAE) was reported by 23 (62.2%) subjects, and 13 (35.1%) subjects had treatment-related AEs (Table 2). The most common TEAEs were headache (13.5%), paresthesia (8.1%), and oral paresthesia (8.1%). There was a slight increase in TEAEs with increasing RI (Table 2). TEAEs were mild in 19 (51.4%) subjects. Two TEAEs moderate in severity (infusion site phlebitis and drug hypersensitivity) were reported in two (5.4%) subjects with mild RI and led to discontinuation of study drug before the end of the IV infusion. There were two serious TEAEs that were considered severe (epiploic appendagitis in a subject with moderate RI and Fournier’s gangrene in a subject with ESRD on HD), both reported as unrelated to study drug and resolved with appropriate medical or surgical intervention. No deaths, treatment-related serious AEs, or TEAEs leading to study discontinuation were reported. No clinically significant abnormalities in the clinical laboratory results, electrocardiogram (ECG) findings, or physical examination were observed. Serum creatinine levels remained stable from screening through the follow-up visit for Cohorts 1–4 (Fig. 1).

**TABLE 2:** Incidence of TEAEs occurring in more than one subject overall

	Number (%) of subjects				
	Cohort 1, normal renal function, eGFR^*a*^ ≥90 mL/min/1.73 m^2^ (*n* = 8)	Cohort 2, mild renalimpairment, eGFR^*a*^ 60–<90 mL/min/1.73 m^2^ (*n* = 8)	Cohort 3, moderate renalimpairment, eGFR^*a*^ 30–<60 mL/min/1.73 m^2^ (*n* = 6)	Cohort 4, severe renalimpairment, eGFR^*a*^<30 mL/min/1.73 m^2^ (*n* = 8)	Cohort 5, end-stage renal disease on hemodialysis(*n* = 7)
Any TEAE	2 (25.0)	7 (87.5)	3 (50.0)	4 (50.0)	7 (100)
Any treatment-related TEAE	1 (12.5)	4 (50.0)	1 (16.7)	3 (37.5)	4 (57.1)
Serious TEAE	0	0	1 (16.7)	0	1 (14.3)
Any TEAE leading to study drug discontinuation	0	2 (25.0)	0	0	0
Catheter site bruise	0	1 (12.5)	0	0	1 (14.3)
Dizziness	0	1 (12.5)	0	1 (12.5)	0
Headache	0	2 (25.0)	1 (16.7)	0	2 (28.6)
Pain in extremity	0	1 (12.5)	0	0	1 (14.3)
Paresthesia	0	1 (12.5)	0	1 (12.5)	1 (14.3)
Paresthesia oral	0	1 (12.5)	0	1 (12.5)	1 (14.3)
Upper respiratory infection	0	2 (25.0)	0	0	0

^*a*^ eGFR, estimated glomerular filtration rate.

### Pharmacokinetics

#### Plasma

**Fig 2:**
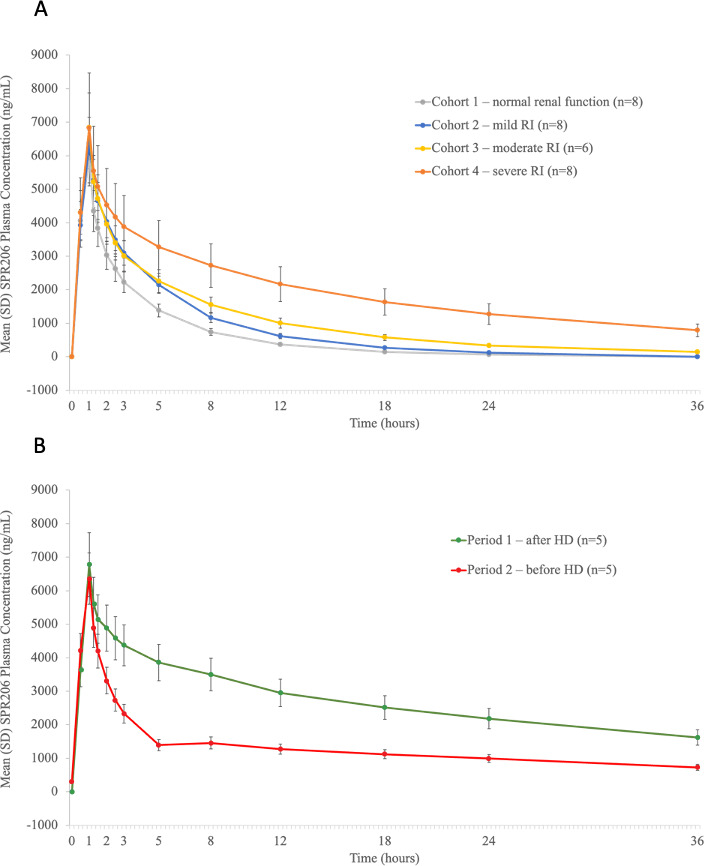
SPR206 plasma concentrations over time for (**A**) Cohorts 1–4 and (**B**) Cohort 5. HD, hemodialysis; RI, renal impairment.

**Fig 3:**
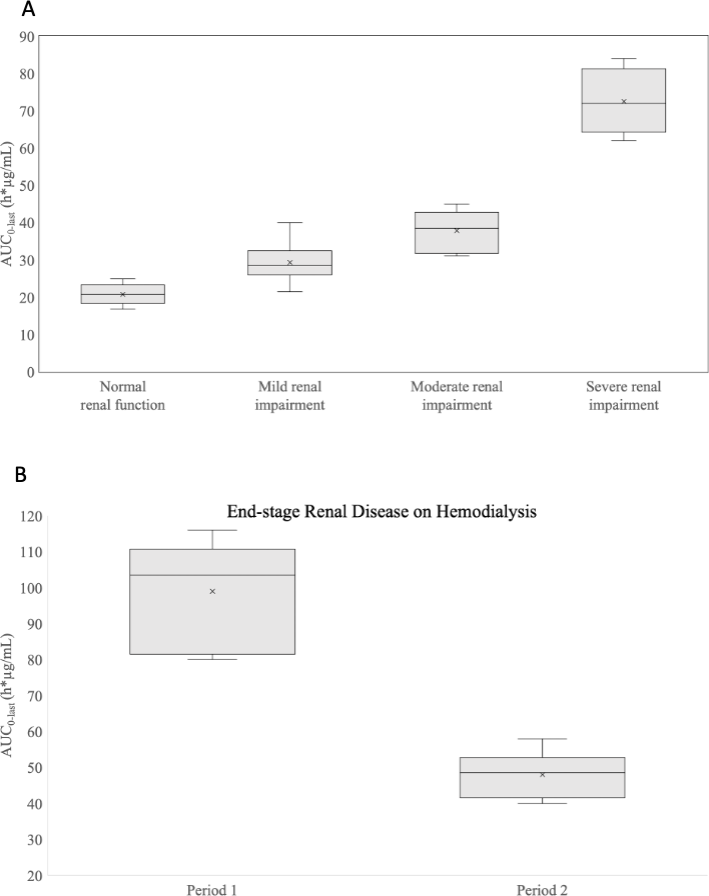
Boxplots for AUC_0–last_ for subjects with normal renal function or varying degrees of renal impairment (**A**) or end-stage renal disease on hemodialysis (**B**) (PK population). Bottom edge, first quartile; middle line, median; top edge, third quartile; X mark, mean; top and bottom whiskers, maximum and minimum values within the upper and lower fence where the fence is 1.5 times the interquartile range from each side of the edge of the box.

**Fig 4:**
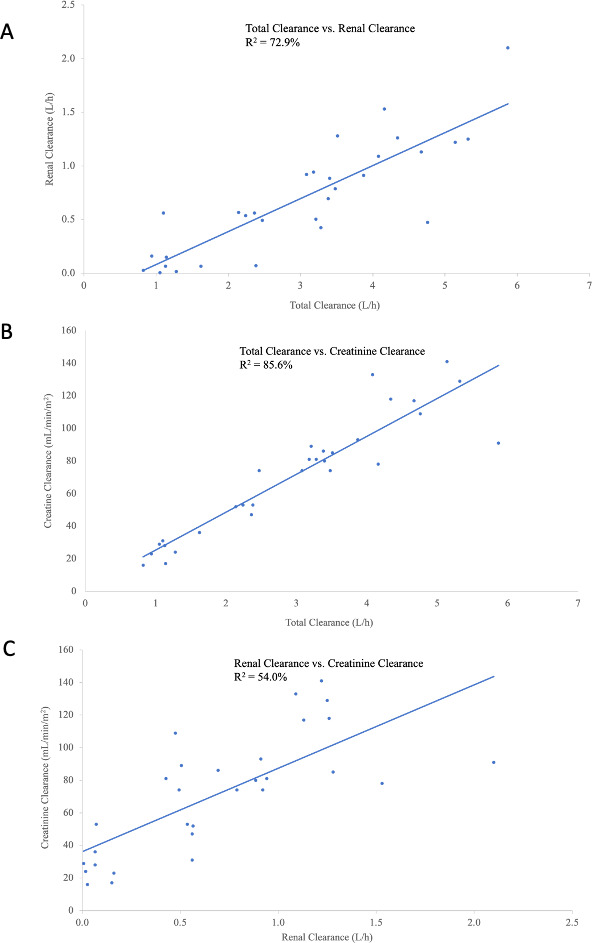
Correlation between (**A**) total clearance and renal clearance, (**B**) total clearance and creatinine clearance, and (**C**) renal clearance and creatinine clearance.

Following a single 100-mg IV dose, SPR206 was rapidly distributed into the systemic circulation across all cohorts. SPR206 plasma concentrations peaked at the end of infusion (1 h) and then declined rapidly except among patients with ESRD dosed after HD (Cohort 5, Period 1) (Fig. 2). Pre-dose concentrations across Cohorts 1–4 were below the limit of quantitation. Mean peak concentrations (*C*_max_) were approximately 6.5 µg/mL for SPR206 across all the cohorts regardless of renal function or timing of HD, while systemic exposure [area under the concentration-time curve (AUC)] increased and clearance (CL) decreased with increasing severity of RI (Table 3). Mean *C*_max_ was similar in subjects with RI and ESRD on HD (Cohorts 2–5) as compared with healthy subjects [least square (LS) geometric mean ratios (GMR) ranged from 1.07 to 1.14] (Table 4). However, mean AUC from time 0 to the last quantifiable concentration (AUC_0–last_) was 39%, 82%, and 239% greater (LS GMR ranged from 1.39 to 3.39) (Fig. 3), and mean AUC extrapolated to infinity (AUC_0–∞_) was 39%, 86%, and 309% greater (LS GMR ranged from 1.39 to 4.09) in subjects with mild, moderate, and severe RI, respectively, compared with healthy subjects. Mean CL was 29%, 46%, and 76% lower (LS GMR ranged from 0.71 to 0.24) in subjects with mild, moderate, and severe RI, respectively, compared with healthy subjects. In subjects with ESRD receiving HD during Period 1 (nondialyzed), mean AUC_0–last_ was 372% (LS GMR of 4.72) greater, and mean CL was 87% lower (LS GMR of 0.13) compared with healthy subjects. In Period 2 (dialyzed), the impact of RI on SPR206 PK was minimized; AUC_0–last_ was 131% greater (LS GMR of 2.31), and CL was 75% lower (LS GMR of 0.25) compared with healthy subjects. Correlations between total CL, renal clearance (CL_R_), and creatinine clearance (CrCL) were linear (Fig. 4).

**TABLE 3:** SPR206 pharmacokinetic parameters^*a*^

Parameter	Cohort 1, normal renal function, eGFR^*b*^ ≥90 mL/min/1.73 m^2^ (*n* = 8)	Cohort 2, mild renalimpairment, eGFR^*b*^ 60–<90 mL/min/1.73 m^2^ *(n* = 8)	Cohort 3, moderate renalimpairment, eGFR^*b*^ 30–<60 mL/min/1.73 m^2^ (*n* = 6)	Cohort 4, severe renalimpairment, eGFR^*b*^<30 mL/min/1.73 m^2^ (*n* = 8)	Cohort 5, end-stage renal disease onhemodialysis Period 1^*c*^ (*n* = 5)	Cohort 5, end-stage renal disease on hemodialysis Period 2^*d*^ (*n* = 5)
*C*_max_ ^*e*^ (µg/mL)	5.9 (11.3)	6.5 (24.5)	6.7 (24.2)	6.7 (18.2)	6.7 (19.8)	6.3 (15.1)
*T*_max_ ^*f*^ (h)	1.0 (1.0, 1.0)	1.0 (1.0, 2.0)	1.0 (1.0, 1.0)	1.0 (1.0, 1.02)	1.0 (1.0, 1.0)	1.0 (1.0, 1.05)
AUC_0–last_ ^*g*^ (µg∙h/mL)	20.7 (13.6)	28.8 (17.0)	37.7 (16.5)	70.1 (22.4)	97.5 (17.7)	47.8 (14.8)
AUC_0–∞_ ^*h*^ (µg∙h/mL)	21.2 (14.1)	29.6 (16.7)	39.5 (17.3)	86.7 (23.9)	Not calculated	Not calculated
*t*_1/2_ ^*i*^ (h)	4.4 ± 0.9	4.9 ± 1.0	8.4 ± 1.4	17.0 ± 4.7	28.1 ± 4.8	32.6 ± 7.7
CL^*j*^ (L/h)	4.7 (14.1)	3.3 (14.5)	2.5 (17.3)	1.1 (20.5)	0.6 (22.9)	1.2 (20.7)
*V*_Z_ ^*k*^ (L)	29.6 (13.9)	23.1 (17.0)	30.4 (18.2)	26.6 (31.3)	24.7 (19.2)	54.4 (7.3)

^*a*^ Geometric mean (coefficient of variation %) is presented for *C*_max_, AUC_0–last_, AUC_0–∞_, CL and *V*_Z_. Arithmetic mean (±standard deviation) is presented for *t*_1/2_. Median (minimum, maximum) is reported for *T*_max_.

^*b*^ eGFR, estimated glomerular filtration rate.

^*c*^ Period 1, the subject was dosed intravenously within 2 h after completion of the scheduled hemodialysis on day 1.

^*d*^ Period 2, the subject was dosed intravenously 1 h prior to the scheduled hemodialysis on day 5.

^*e*^ *C*_max_, maximum plasma concentration.

^*f*^ *T*_max_, time to peak concentration.

^*g*^ AUC_0–last_, area under the concentration-time curve from time 0 to last measurable time point.

^*h*^ AUC_0–∞_, area under the concentration-time curve from time 0 to infinity.

^*i*^ *t*_1/2_, elimination half-life.

^*j*^ CL, total body clearance.

^*k*^ *V*_Z_, volume of distribution at the terminal phase.

**TABLE 4:** Comparison of plasma pharmacokinetic parameters between Cohorts 2–5 and Cohort 1^*a*^

Parameter	Statistic	Cohort 1, normal renal function, eGFR^*b*^ ≥90 mL/min/1.73 m^2^ *(n* = 8)	Cohort 2, mild renalimpairment, eGFR^*b*^ 60–<90 mL/min/1.73 m^2^ (*n* = 8)	Cohort 3, moderate renalimpairment, eGFR^*b*^ 30–<60 mL/min/1.73 m^2^ (*n* = 6)	Cohort 4, severe renalimpairment, eGFR^*b*^<30 mL/min/1.73 m^2^ (*n* = 8)	Cohort 5, end-stage renal disease on hemodialysis Period 1^*c*^ (*n* = 5)	Cohort 5, end-stage renal disease on hemodialysis Period 2^*d*^ (*n* = 5)
*C*_max_ ^*e*^(µg/mL)	LS GM (SE)^*f*^	5.9 (0.068)	6.5 (0.068)	6.7 (0.078)	6.7 (0.068)	6.7 (0.086)	6.3 (0.086)
	GMR^*g*^		1.09 (0.93, 1.29)	1.13 (0.95, 1.35)	1.14 (0.97, 1.34)	1.13 (0.94, 1.36)	1.07 (0.89, 1.29)
AUC_0–8_ ^*h*^(µg∙h/mL)	LS GM (SE)^*f*^	16.7 (0.055)	21.8 (0.055)	22.8 (0.064)	28.7 (0.055)	32.4 (0.070)	19.0 (0.070)
	GMR^*g*^		1.31 (1.14, 1.49)	1.37 (1.18, 1.58)	1.73 (1.51, 1.97)	1.95 (1.67, 2.26)	1.14 (0.98, 1.33)
AUC_0–last_ ^*i*^(µg∙h/mL)	LS GM (SE)^*f*^	20.7 (0.061)	28.8 (0.061)	37.7 (0.071)	70.1 (0.061)	97.5 (0.077)	47.8 (0.077)
	GMR^*g*^		1.39 (1.20, 1.61)	1.82 (1.56, 2.13)	3.39 (2.93, 3.92)	4.72 (3.99, 5.57)	2.31 (1.95, 2.73)
AUC_0–∞_ ^*j*^ (µg∙h/mL)	LS GM (SE)^*f*^	21.2 (0.060)	29.6 (0.060)	39.5 (0.070)	86.7 (0.086)	Not calculated	Not calculated
	GMR^*g*^		1.39 (1.20, 1.62)	1.86 (1.59, 2.18)	4.09 (3.42, 4.89)	Not calculated	Not calculated
CL^*k*^ (L/h)	LS GM (SE)^*f*^	4.7 (0.063)	3.3 (0.063)	2.5 (0.073)	1.1 (0.063)	0.6 (0.080)	1.2 (0.080)
	GMR^*g*^		0.71 (0.61, 0.82)	0.54 (0.46, 0.63)	0.24 (0.20, 0.28)	0.13 (0.11, 0.16)	0.25 (0.21, 0.30)

^*a*^ Least square geometric mean ratio [90% confidence interval (CI)] for each cohort was computed with respect to Cohort 1. Natural log transformation was applied to the pharmacokinetic parameter data prior to analysis. The analysis of variance model included the pharmacokinetic parameter as the response variable with the cohort as fixed factor taking values as 01 to 04 for Cohorts 1–4 and 05 and 06 for Cohort 5 Period 1 and Cohort 5 Period 2, respectively. Estimated mean difference and associated 90% CI was back transformed to provide geometric mean ratios and CIs.

^*b*^ eGFR, estimated glomerular filtration rate.

^*c*^ Period 1, the subject was dosed intravenously within 2 h after completion of the scheduled hemodialysis on day 1.

^*d*^ Period 2, the subject was dosed intravenously 1 h prior to the scheduled hemodialysis on day 5.

^*e*^ *C*_max_, maximum plasma concentration.

^*f*^ LS GM (SE), least square geometric mean (standard error).

^*g*^ GMR, geometric mean ratio.

^*h*^ AUC_0–8_, area under the concentration-time curve from time 0 to 8 h.

^*i*^ AUC_0–last_, area under the concentration-time curve from time 0 to last measurable time point.

^*j*^ AUC_0–∞_, area under the concentration-time curve from time 0 to infinity.

^*k*^ CL, total body clearance.

In those with ESRD (Cohort 5), HD had little effect on the mean *C*_max_ of SPR206 (LS GMR of approximately 1) (Table 5). However, mean AUC_0–last_ was 51% lower (LS GMR of 0.49), and mean CL was approximately 92% greater (LS GMR of 1.92) in Period 2 (dialyzed) compared with Period 1 (nondialyzed). No relationship was observed between *C*_max_ and estimated glomerular filtration rate (eGFR) across cohorts (*P* = 0.123), while AUC was negatively correlated and CL positively correlated with eGFR (*P* < 0.0001) (Table 6).

**TABLE 5:** Effect of hemodialysis on SPR206 plasma pharmacokinetic parameters in subjects from Cohort 5^*a*^

Parameter	Statistic	Cohort 5,end-stage renaldisease onhemodialysis,Period 1^*b*^ (*n* = 5)	Cohort 5, end-stage renal disease onhemodialysis, Period 2^*c*^ (*n* = 5)
AUC_0−8_ ^*d*^ (µg∙h/mL)	LS GM (SE)^*e*^	29.6 (0.072)	17.3 (0.072)
	LS GMR^*f*^ (90% CI)	0.59 (0.52, 0.67)	
AUC_0−last_ ^*g*^ (µg∙h/mL)	LS GM (SE)^*e*^	91.5 (0.099)	44.8 (0.099)
	LS GMR^*f*^ (90% CI)	0.49 (0.39, 0.62)	
*C*_max_ ^*h*^ (µg/mL)	LS GM (SE)^*e*^	6.0 (0.059)	5.7 (0.059)
	LS GMR^*f*^ (90% CI)	0.94 (0.82, 1.08)	
CL^*i*^ (L/h)	LS GM (SE)^*e*^	0.7 (0.113)	1.4 (0.113)
	LS GMR^*f*^ (90% CI)	1.92 (1.58, 2.34)	

^*a*^ Natural log transformation was applied to the pharmacokinetic parameter data prior to analysis. The mixed model included pharmacokinetic parameter as the response variable with period as fixed factor, subject as random factor and body weight at baseline, age, and sex as covariates. Estimated mean difference and associated 90% CI was back transformed to provide geometric mean ratios and CIs. AUC_0−∞_, area under the concentration-time curve from time 0 to infinity, was not calculable.

^*b*^ Period 1, the subject was dosed intravenously within 2 h after completion of the scheduled HD on day 1.

^*c*^ Period 2, the subject was dosed intravenously 1 h prior to the scheduled HD on day 5.

^*d*^ AUC_0−8_, area under the concentration-time curve from time 0 to 8 h.

^*e*^ LS GM (SE), least square geometric mean (standard error).

^*f*^ LS GMR, least square geometric mean ratio.

^*g*^ AUC_0−last_, area under the concentration-time curve from time 0 to last measurable time point.

^*h*^ *C*_max_, maximum plasma concentration.

^*i*^ CL, total body clearance.

**TABLE 6:** Relationship between renal function and pharmacokinetic parameters (PK population)^*a*^

Dependent variable	Statistic	Cohorts 1–5 (*N* = 35)
*C*_max_ ^*b*^ (µg/mL)	Intercept	2.0
	Standard error	0.0011
	Coefficient (95% CI)	−0.0018 (−0.0040, 0.0005)
	*P*-value	0.123
	R^2^	0.083
AUC_0-8_ ^*c*^ (µg∙h/mL)	Intercept	3.5
	Standard error	0.001
	Coefficient (95% CI)	−0.0063 (−0.0083, –0.0044)
	*P*-value	<0.0001
	R^2^	0.611
AUC_0-last_ ^*d*^ (µg∙h/mL)	Intercept	4.5
	Standard error	0.001
	Coefficient (95% CI)	−0.0147 (−0.0168, –0.0125)
	*P*-value	<0.0001
	R^2^	0.876
AUC_0-∞_ ^*e*^ (µg∙h/mL)	Intercept	4.6
	Standard error	0.0014
	Coefficient (95% CI)	−0.0161 (−0.0190, –0.0131)
	*P*-value	<0.0001
	R^2^	0.839
CL^*f*^ (L/h)	Intercept	0.1
	Standard error	0.0012
	Coefficient (95% CI)	0.0172 (0.0148, 0.0196)
	*P*-value	<0.0001
	R^2^	0.888

^*a*^ The relationship between dependent variables (i.e., AUC_0−8_, AUC_0−last_, AUC_0−∞_, *C*_max_, and clearance) and the independent variable (i.e., estimated glomerular filtration rate) was quantified by fitting a simple linear regression model, respectively.

^*b*^ *C*_max_, maximum plasma concentration.

^*c*^ AUC_0–8_, area under the concentration-time curve from time 0 to 8 h.

^*d*^ AUC_0−last_, area under the concentration-time curve from time 0 to last measurable time point.

^*e*^ AUC_0–∞_, area under the concentration-time curve from time 0 to infinity.

^*f*^ CL, total body clearance.

#### Urine

**Fig 5:**
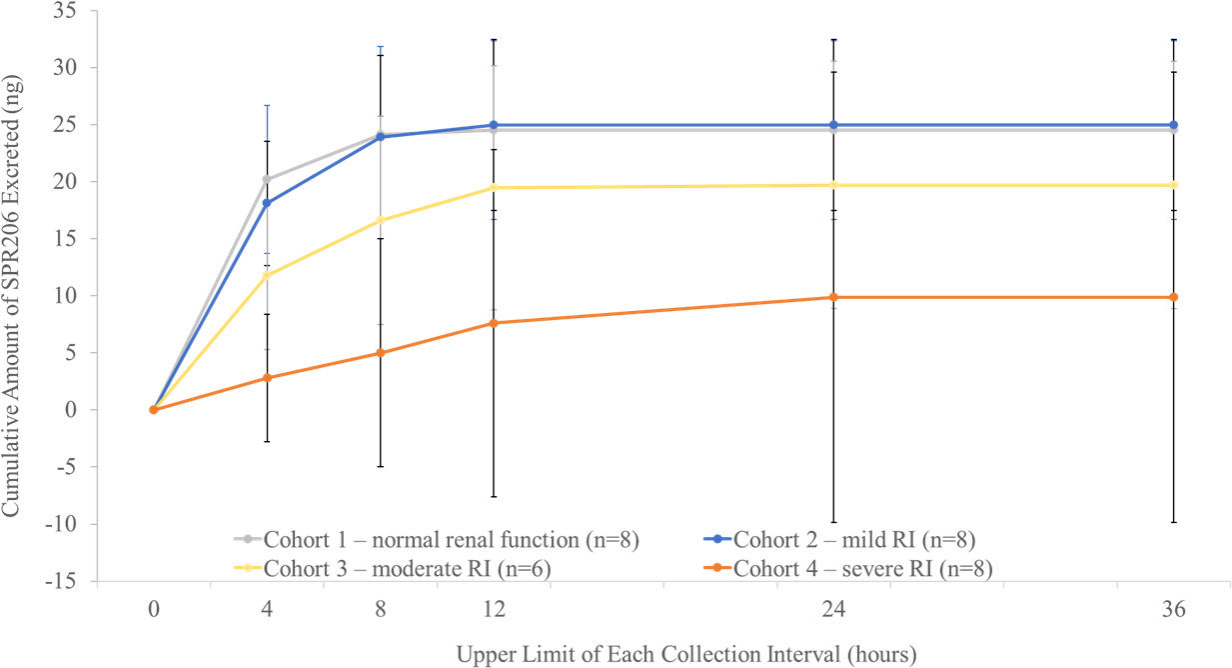
Mean cumulative amount of SPR206 excreted unchanged in urine for Cohorts 1–4. RI, renal impairment.

Mean cumulative urine concentrations of SPR206 excreted over time decreased with increasing severity of RI (Fig. 5). Mean concentrations were similar in healthy subjects and subjects with mild RI and were below the limit of quantitation (BLQ <100 ng/mL) after 12 h for most of these subjects, indicating complete excretion within 12 to 24 h after dosing. In subjects with moderate or severe RI, urine SPR206 concentrations were quantifiable up to 36 h, suggesting prolonged excretion, and were consistently lower across this time period than in healthy subjects or subjects with mild RI. Mean CL_R_ for SPR206 decreased with increasing severity of RI, with mean values highest (1.18 L/h) in healthy subjects and lowest (0.13 L/h) in subjects with severe RI (Cohort 4) (Table 7). Mean CL_R_ was 24.6% of total CL in healthy subjects, indicating that renal elimination is an important route of excretion. In Cohorts 2, 3, and 4, CL_R_ was 25.6%, 20.5%, and 12.0% of total CL, respectively. Mean cumulative amount of SPR206 excreted in urine (Aeu) and mean cumulative fraction of dose excreted in the urine (Feu) over the 24- to 36-h urine collection interval values were similar in healthy subjects (Cohort 1) and subjects with mild RI (Cohort 2). Mean amounts of SPR206 and mean fractions of the SPR206 dose excreted unchanged in urine (by interval, Aet; expressed as a percentage, Ae%; and as a fraction over a collection interval, Fe) were consistently greater in Cohorts 1 and 2 compared with Cohorts 3 and 4.

**TABLE 7:** Geometric mean and geometric coefficient of variation (%) for SPR206 urine pharmacokinetic parameters in Cohorts 1–4^*a*^

Parameter	Timepoint, post-infusion (h)	Cohort 1, normal renal function, eGFR^*b*^ ≥90 mL/min/1.73 m^2^ (*n* = 8)	Cohort 2, mild renal impairment, eGFR^*b*^ 60 to <90 mL/min/1.73 m^2^ (*n* = 8)	Cohort 3, moderate renal impairment, eGFR^*b*^ 30 to <60 mL/min/1.73 m^2^ (*n* = 6)	Cohort 4, severe renal impairment, eGFR^*b*^<30 mL/min/1.73 m^2^ (*n* = 8)
CL_R_ ^*c*^ (L/h)	0–36	1.100 (43.4)	0.811 (45.7)	0.421 (111.5)	0.059 (262.5)
Aeu^*d*^ (mg)	0–36	23.240 (38.9)	23.829 (35.4)	15.860 (111.3)	4.158 (282.3)
Feu^*e*^	0–36	0.232 (38.8)	0.242 (36.1)	0.159 (110.5)	0.041 (285.7)
Aet^*f*^ (mg)	0–4	18.890 (44.2)	17.380 (34.6)	7.965 (224.7)	0.729 (2566.2)
	4–8	3.058 (90.8)	4.829 (66.2)	3.372 (148.0)	0.648 (389.0)
	8–12	0.467 (88.7)	0.613 (219.2)	1.507 (93.8)	0.450 (283.6)
	12–24	0.092	0.434	0.587 (171.9)	1.108 (295.1)
	24–36	Not calculated	Not calculated	1.490	0.374 (607.4)
Ae%^*g*^	0–4	81.281 (14.4)	72.948 (15.7)	50.180 (53.6)	17.679 (309.1)
	4–8	13.150 (84.7)	20.260 (46.3)	21.268 (89.3)	15.576 (37.7)
	8–12	2.019 (96.2)	2.574 (175.5)	9.505 (82.5)	10.834 (65.8)
	12–24	0.346	1.680	3.699	26.651
	24–36	Not calculated	Not calculated	9.640	7.821 (33.7)
Fe^*h*^	0–4	0.189 (44.1)	0.176 (34.4	0.079 (236.9)	0.015 (325.4)
	4–8	0.031 (89.5)	0.049 (68.3)	0.034 (150.9)	0.010 (230.1)
	8–12	0.005 (102.7)	0.009 (86.6)	0.015 (89.6)	0.004 (301.1)
	12–24	0.001	0.004	0.006 (151.3)	0.011 (283.8)
	24–36	Not calculated	Not calculated	0.015	0.007 (299.6)

^*a*^ Aeu, Feu, and CL_R_ results reported over the 0- to 36-h collection interval.

^*b*^ eGFR, estimated glomerular filtration rate.

^*c*^ CL_R_, renal clearance.

^*d*^ Aeu, cumulative amount of drug excreted in urine at the end of each interval.

^*e*^ Feu, cumulative fraction of dose excreted in the urine over a collection interval.

^*f*^ Aet, amount of drug excreted in urine by interval.

^*g*^ Ae%, fraction of drug excreted in the urine expressed as a percentage.

^*h*^ Fe, fraction of dose excreted in the urine over a collection interval.

## DISCUSSION

This Phase 1 study evaluated the safety, tolerability, and PK of SPR206 in healthy subjects with normal renal function and subjects with varying degrees of RI or ESRD on HD. The study was designed in accordance with guidelines from the Food and Drug Administration for assessing the influence of RI on the PK of investigational drugs (41). A single IV dose of SPR206 100 mg was generally safe and well tolerated in healthy subjects, subjects with varying degrees of RI, and subjects with ESRD on HD. The most common TEAEs were headache and paresthesia, which were mostly mild in severity.

After a single IV dose, systemic exposure to SPR206 increased as the RI increased, while mean plasma CL decreased. Analysis of the effect of HD on the PK of SPR206 in subjects with ESRD found a decrease in systemic exposure and an increase in plasma CL with dialysis, as expected. A significant negative correlation between eGFR and AUC and a significant positive correlation between eGFR and total CL were observed. SPR206 was nearly completely excreted in urine within 12 h in healthy subjects and those with mild RI, but urinary excretion was slower in subjects with moderate and severe RI. These findings may be contrasted with available data for colistin and polymyxin B, although dose adjustment recommendations are based on limited published data. While colistin and polymyxin B appear to be eliminated primarily by non-renal mechanisms (42), the prodrug colistin methanesulfonate (CMS) is eliminated predominantly by renal excretion (42, 43). Thus, as CrCL declines, elimination of CMS decreases. Based on expert recommendations, the dose of colistin should be adjusted in HD, while the dose of polymyxin B is not significantly affected by HD (44).

The PK findings in the healthy subject cohort of this study are consistent with previous findings from Phase 1 studies of SPR206 in healthy subjects (40, 45). After a single 100-mg IV dose of SPR206, *C*_max_ was 5.3 µg/mL, AUC_0–∞_ was 20.4 h·μg/mL, and total CL was 5.0 L/h, which were comparable to PK findings in this study (40). In a second study that investigated pulmonary concentrations of SPR206 in healthy subjects, *C*_max_ was 4.4 µg/mL and AUC from time 0 to 8 h (AUC_0–8_) was 20.1 h·μg/mL (45). Protein binding was reported to be ≤12.8% in humans with a molecular weight of 1145 Da (data on file, Spero Therapeutics, Inc.).

Current treatment options for serious infections caused by MDR Gram-negative bacteria include carbapenems, carbapenem/beta-lactamase inhibitor combinations, siderophore cephalosporins, and tetracyclines. However, the use of concomitant aminoglycosides and polymyxins with these agents continues for serious life-threatening infections despite limitations of nephrotoxicity, neurotoxicity, and ototoxicity (25, 26, 46 – 48). Therapeutic drug monitoring is required with the use of aminoglycosides (49), and older polymyxins may be associated with acute kidney injury in up to 60% of patients (44).

The development of SPR206, which is a novel polymyxin B analog with a β-branched aminobutyrate N-terminus and an aryl substituent, was the result of an effort to reduce the nephrotoxicity potential compared to polymyxins (38) and to maintain the *in vitro* and *in vivo* activity of polymyxins against MDR Gram-negative pathogens. In rat models, SPR206 demonstrated lower kidney cell cytotoxicity and lower kidney exposure than polymyxin B (38, 39). Results from preclinical studies and Phase 1 studies with SPR206 suggest the potential of broad spectrum of activity against MDR Gram-negative pathogens causing serious infections, which is at least comparable to polymyxins but with a clinically relevant improvement in the safety profile.

In summary, this study characterized the safety, tolerability, and PK of SPR206 in subjects with varying degrees of RI. Because AUC_0–∞_ could not be accurately estimated in Cohort 5, AUC_0–last_ was utilized, which likely underestimates AUC, and the GMR ratios relative to healthy subjects are likely higher than reported. As expected for a renally eliminated drug, plasma AUC for SPR206 increased with decreasing renal function. Based on these results, a reduced dosage of SPR206 may be needed in patients with severe RI and in patients with ESRD on HD. Importantly, the safety and tolerability profile of SPR206 in subjects with varying degrees of RI were consistent with matched healthy subjects in this study and healthy subjects from the previous single-ascending and multiple-ascending dose study (40). The slight increase in TEAEs associated with increasing RI is consistent with this population including subjects with ESRD. Incorporating these data into a population PK model and utilizing pharmacokinetic-pharmacodynamic target attainment analyses will assist in developing dosing recommendation for patients with varying degrees of RI, including ESRD on HD.

## MATERIALS AND METHODS

The study was conducted between June 2020 and December 2022 at New Zealand Clinical Research, Christchurch and Auckland, New Zealand, in accordance with the US Code of Federal Regulations and ethical principles of the Declaration of Helsinki, Good Clinical Practices, and International Council for Harmonisation guidelines. The study protocol and all amendments were reviewed by an institutional review board for the two study centers (Health and Disability Ethics Committees, Ministry of Health, Wellington, New Zealand). Informed consent was obtained from each subject in writing before any study procedures were performed. This study was registered at clinicaltrials.gov NCT04865393.

### Study design

This was a Phase 1, open-label study to assess the safety, tolerability, and PK of a single 100-mg IV dose of SPR206 administered to healthy adults with normal renal function and those with varying degrees of RI including subjects with ESRD on HD. Subjects were screened within 28 days prior to dosing. Study drug administration occurred on day 1 for Cohorts 1–4 (normal renal function, mild RI, moderate RI, and severe RI) and on days 1 and 5 for Cohort 5 (ESRD on HD). Subjects remained confined to the clinical study unit from day −1 (1 day prior to study drug administration) until completion of the scheduled study procedures at the end of confinement on day 4 (Cohorts 1–4) or on day 7 (Cohort 5). A follow-up safety visit occurred 7 to 14 calendar days after the last dose for all subjects.

Subjects were categorized into Cohorts 1–4 at screening using eGFR, which was calculated using the Modification of Diet in Renal Disease (50) equation. Subjects with ESRD on HD were assigned to Cohort 5 at screening. Estimated CrCL using the Cockcroft-Gault equation (51) was calculated for subjects in Cohorts 1–4 at screening. Initially, the study allowed simultaneous enrollment in Cohorts 2–5. After enrollment of at least 50% of subjects in Cohorts 3 and 4, matched controls were enrolled in Cohort 1.

On the morning of day 1, subjects in Cohorts 1–4 received a single 100-mg IV dose of SPR205 infused over 1 h. Subjects in Cohort 5 received a single IV dose of SPR206 within 2 h (±1 h) after completion of regularly scheduled HD on day 1 (Period 1) and a second dose approximately 1 h prior to their regularly scheduled HD on day 5 (Period 2). Details of the HD procedure are provided in the Supplement.

### Subject selection

Adult men or women at least 18 years of age were eligible if they had a BMI ≥18.5 and ≤39.9 kg/m^2^ and body weight between 50.0 and 130.0 kg inclusive. Subjects had to be medically healthy without clinically significant abnormalities (Cohort 1 only) or medically stable without clinically significant acute or chronic illness (Cohorts 2–5) that could impact the assessment of safety and PK based on screening medical history, physical examination, vital signs, 12-lead ECG, and clinical laboratory testing. Subjects in Cohort 1 had normal renal function (eGFR ≥90 mL/min/1.73 m^2^), Cohort 2 had an eGFR of 60 to <90 mL/min/1.73 m^2^, Cohort 3 had an eGFR of 30 to <60 mL/min/1.73 m^2^, and Cohort 4 had an eGFR <30 mL/min/1.73 m^2^. Subjects in Cohort 5 with ESRD were receiving HD at least three times per week for at least 3 months at screening. Women had to be non-pregnant and non-lactating and, if not postmenopausal, were required to use an acceptable form of contraception throughout the study and for 30 days after completion. Matched controls were based on a rolling pooled mean for BMI (± 20%), sex (similar ratio 1:1 ± 1), and mean age (−25/+10 years) observed in subjects in Cohorts 3 and 4.

### Study assessments

Study assessments included a complete physical examination, vital signs (blood pressure, heart rate, respiratory rate, and body temperature), 12-lead ECG, clinical laboratory tests (hematology, blood chemistry, coagulation, and urinalysis), monitoring of AEs, and PK samplings.

Subjects in Cohorts 1–4 had serial blood samples collected to determine plasma SPR206 concentrations at pre-dose (0) and 0.5, 1, 1.25, 1.5, 2, 2.5, 3, 5, 8, 10, 12, 18, 24, and 36 h post-dose. Total voided urine was collected over a 2-h interval from −2 to 0 h (pre-dose) and at 0–4, 4–8, 8–12, 12–24, and 24–36 h post-dose. For subjects in Cohort 5, blood samples were collected on dosing days 1 and 5 pre-dose (0 h) and 0.5, 1, 1.25, 1.5, 2, 2.5, 3, 5, 8, 10, 12, 18, 24, and 36 h after dosing. On day 5 (Period 2), the 1-h post-dose sample was collected immediately prior to initiation of dialysis, and the 5-h post-dose sample was collected 10 min prior to the end of dialysis. The 36-h post-dose samples on days 3 and 7 were collected prior to the next dialysis session if scheduled on the same day. In Period 2, blood samples were collected from both the inflow (arterial) and outflow (venous) lines pre-dialysis and at approximately 1, 2, 3, and 4 h after initiation of HD on day 5. If the HD session was shorter or longer than 4 h after the start of HD, a final sample was collected at the end of dialysis. Plasma and urine samples were assayed for SPR206 using a validated liquid chromatography tandem mass spectrometry method (QPS, LLC, Newark, DE, USA). For plasma, the assay range was 50 to 50,000 ng/mL, and for urine, the range was 100 to 50,000 ng/mL. In plasma, the intraday precision (% coefficient of variation) ranged from 1.3% to 7.7%, and the accuracy (% relative error) ranged from 1.6% to 6.0%. In urine, the intraday precision (% coefficient of variation) ranged from 1.7% to 7.5%, and the accuracy (% relative error) ranged from −4.8% to 7.3%. Additional details are provided in the Supplement.

### Pharmacokinetic analysis

For all cohorts, the following PK parameters were calculated using noncompartmental methods based on plasma SPR206 concentrations: *C*_max_, *T*_max_, AUC_0−last_, AUC_0−8_, AUC_0−∞_, terminal elimination half-life (*t*_1/2_), CL, and volume of distribution at the terminal phase (*V*_Z_). For Cohort 5, additional parameters were extraction ratio (ER), estimated hemodialysis clearance, and amount of the dose removed by hemodialysis. The ER was calculated as 100 × [(CA − CV)/CA], where CA and CV were predialyzer and postdialyzer paired drug concentrations at the arterial and venous sites. SPR206 clearance during HD (CL_HD_) was calculated using the following equation: CL_HD_ = Q × ER, where Q was the known blood flow through the dialyzer. The amount of SPR206 removed by dialysis (X_HD_) was estimated by multiplying the AUC during dialysis [i.e., AUC from the beginning of dialysis to end of dialysis (AUC_on–HD_)] by CL_HD_: X_HD_ = AUC_on–HD_ × CL_HD_.

Urine PK parameters included Aet, Ae%, Aeu, CL_R_, Fe, and Feu.

To model the relationship between renal function and PK parameters, a linear regression analysis was performed using log-transformed values of AUC_0–8_, AUC_0–last_, AUC_0–∞_, *C*_max_, and CL, which were dependent variables. The independent variable to predict the PK parameters was eGFR after adjusting for baseline covariates (age, gender, race, and body weight). Parameter estimates, standard errors, confidence intervals (CIs), and *P*-values were presented.

All BLQ values (<100 ng/mL) or missing values were reported. All PK evaluations were performed using Phoenix WinNonlin version 8.3 (Pharsight Corporation, Mountain View, CA, USA).

### Statistical analysis

No formal sample size calculation was performed. A sample size of eight subjects per cohort was considered sufficient to provide adequate data for inclusion in the PK analysis. Eight subjects were recruited in each cohort to ensure at least six evaluable subjects per cohort. Subjects who did not complete critical study procedures could be replaced.

Mean and individual plasma concentration-time curves were tabulated for each cohort (and for Cohort 5 subjects by period). Pharmacokinetic parameters were determined for each subject and summarized by cohort (and for Cohort 5 subjects by period) using descriptive statistics (arithmetic mean, SD, coefficient of variation, minimum, maximum, and median). In addition, geometric means were calculated for AUC and *C*_max_.

For estimation of PK parameters for RI subjects (Cohorts 2–5) compared to healthy subjects, an analysis of variance model was used with log-transformed values for AUC_0–8_, AUC_0–last_, AUC_0–∞_, *C*_max_, and CL as the response variables and with the fixed-effect term of cohort as a categorical variable. The estimated LS mean difference and associated 90% CI were calculated for each RI group vs healthy subjects and then back transformed to provide geometric LS mean ratios and 90% CIs for each comparison.

In addition, Periods 1 and 2 for Cohort 5 were compared to assess the effect of HD. For this analysis, data for Cohort 5 from both Period 1 and Period 2 (before HD) were used and were considered separately in the categorical analysis. To evaluate the effect of dialysis on SPR206, AUC_0–8_, AUC_0–last_, AUC_0–∞_, *C*_max_, and CL obtained with dosing before HD (dialyzed) vs dosing after HD (nondialyzed) were compared. The log-transformed values in Period 2 (dialyzed PK) vs Period 1 (nondialyzed PK) were evaluated using a mixed-effect model with period as the factor, body weight at baseline, age, and sex as covariates and subject as the random effect. The two-sided 90% CIs for the estimated ratio of the effect of dialysis (using the period effect) were calculated for AUC_0–8_, AUC_0–last_, AUC_0–∞_, *C*_max_, and CL. The ratio of the geometric means and their CI was obtained by back transforming the estimated mean difference and its corresponding CI. All statistical evaluations were conducted using SAS version 9.4.

## SUPPLEMENTAL MATERIAL

Supplement aac.00505-23-s0001.pdf Individual SPR206 plasma concentrations vs. time for each cohort, and plasma and urine assays.

ASM does not own the copyrights to Supplemental Material that may be linked to, or accessed through, an article. The authors have granted ASM a non-exclusive, world-wide license to publish the Supplemental Material files. Please contact the corresponding author directly for reuse.
