# Supplementary material for: Safety and pharmacokinetics of SPR206 in subjects with varying degrees of renal impairment
Source: Antimicrob Agents Chemother. 2023 Oct 12;67(11):e00505-23. doi: 10.1128/aac.00505-23 (PMC10648942; doi:10.1128/aac.00505-23)
Supplement: Supplement — Individual SPR206 plasma concentrations vs. time for each cohort, and plasma and urine assays. [file aac.00505-23-s0001.pdf]

Supplement

Figure. Individual SPR206 plasma concentrations vs. time for each cohort.

Cohort 1

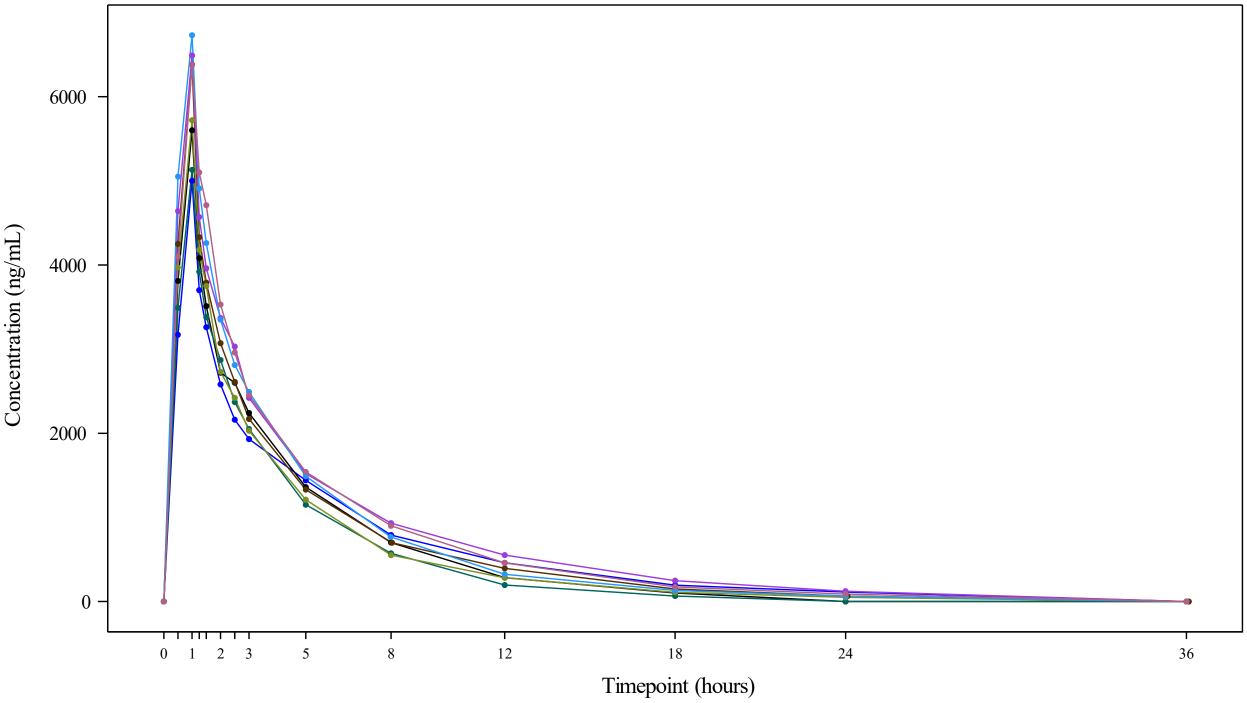

Cohort 2

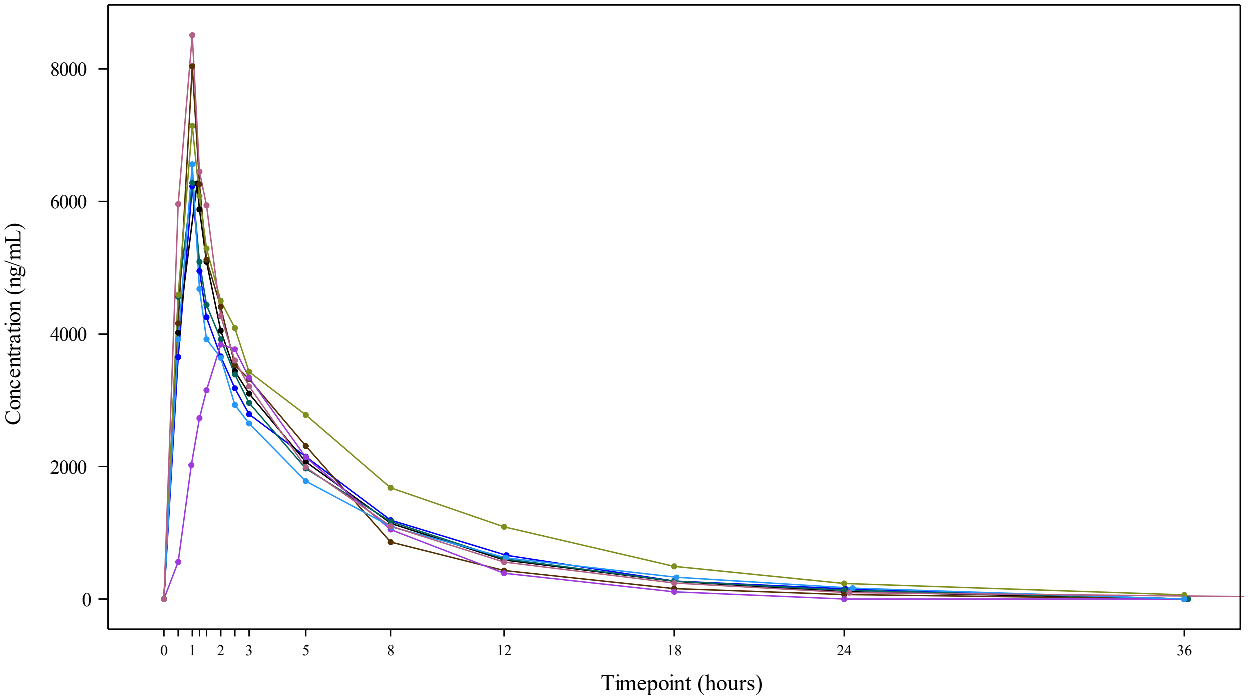

Cohort 3

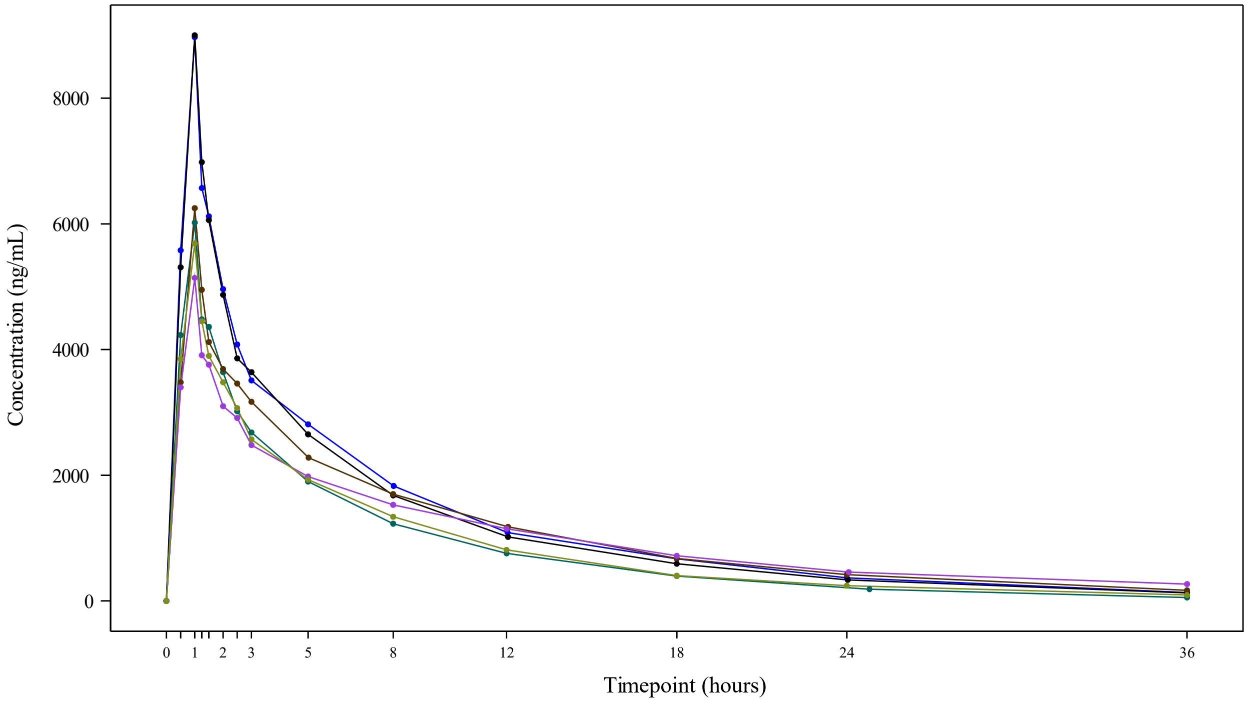

Cohort 4

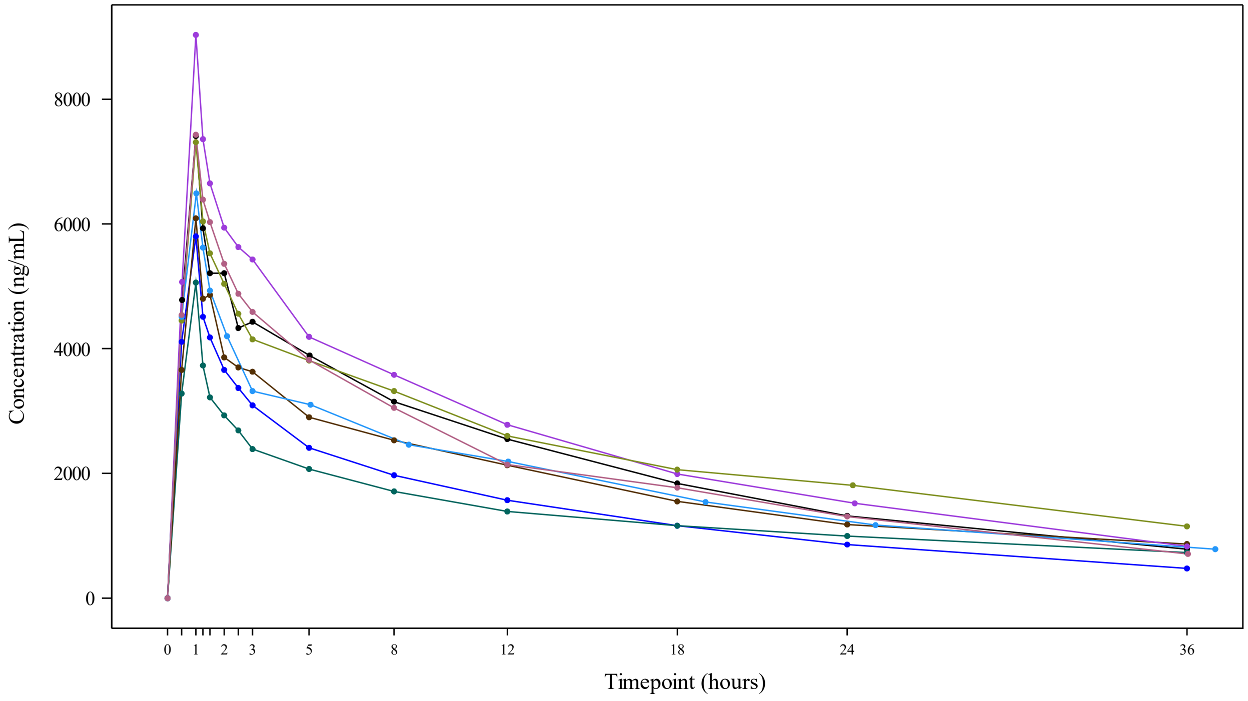

Cohort 5, Period 1

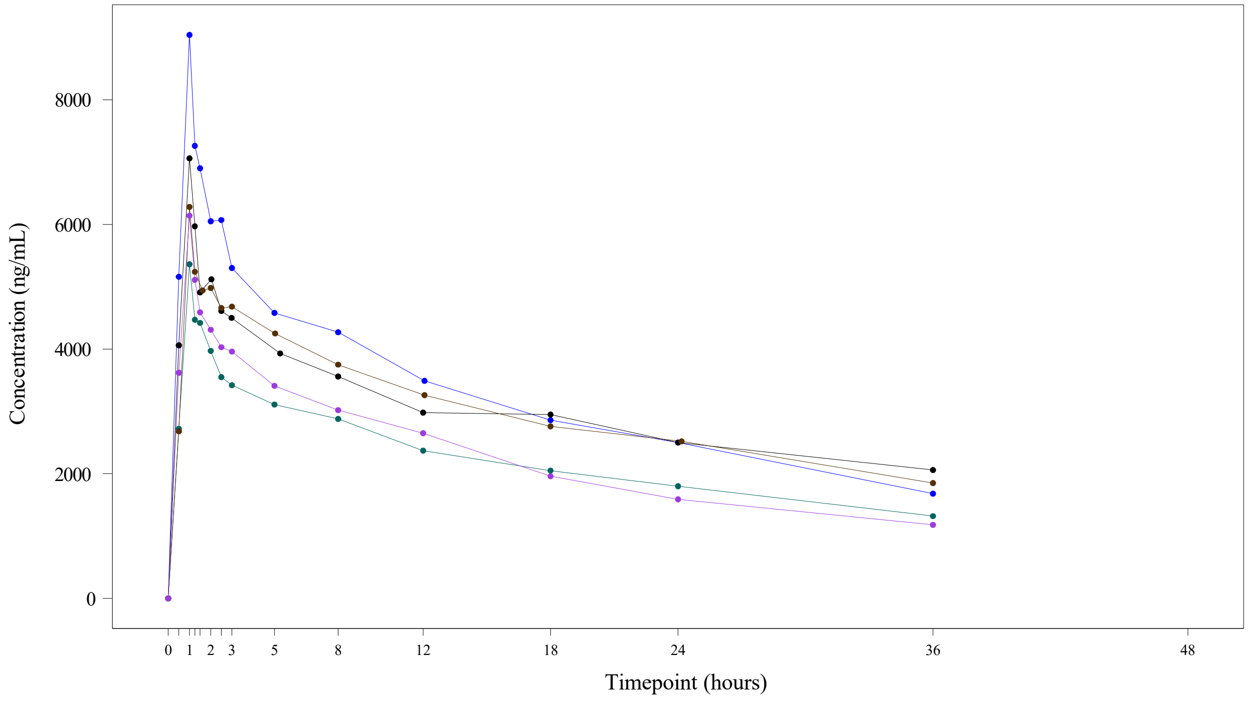

Cohort 5, Period 2

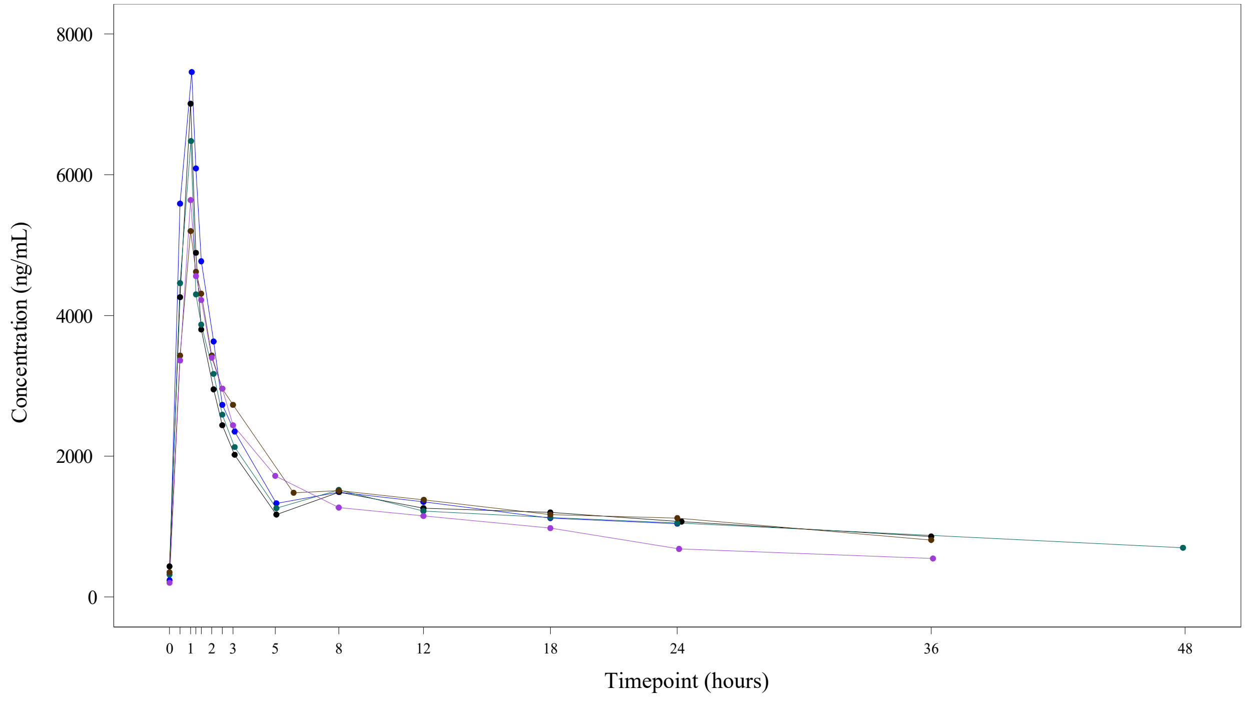

Human Plasma Assay

Method Performance Summary

Assay Passing Rate	SPR206: 10 of 10 runs (100.0%) met acceptance criteria, including ISR runs.
Standard Calibration Curve	SPR206: Eight calibration standards from 50 to 50,000 ng/mL.
Standard Calibration Performance	SPR206: Cumulative bias range: -3.4% to 2.7%
	SPR206: Cumulative precision: ≤7.0% CV
QC Performance	SPR206: Cumulative bias range: 1.6% to 13.0%
	SPR206: Cumulative precision: ≤6.6% CV
Method Reproducibility	SPR206: Incurred sample reanalysis was performed in 11.6% of analyzed study samples, and 100.0% met the pre-specified acceptance criteria.

Sample Storage Conditions

Samples were received at QPS frozen on dry ice, in good condition, and upon arrival they were stored in a -70°C freezer.

Sample Collection

Blood was collected from all subjects in on Day 1 and on Day 5 (Cohort 5 only) into tubes containing K2EDTA as the anticoagulant at the following timepoints: prior to dosing and at 0.5, 1, 1.25, 1.5, 2, 2.5, 3, 5, 8, 2, 18, , 24 and 36 hours post-dose. Arterial and venous blood samples were also collected from Cohort 5 at the following time points: prior to dialysis, and at 1, 2, 3, and 4 hours post-dialysis. Samples were collected from 11 Jun 2021 through 01 Dec 2021.

Sample Inventory

Of the 689 primary samples received, 112 samples were not analyzed because they thawed during shipping. Therefore, their corresponding backup samples were analyzed instead. One primary sample was not analyzed because the tube label was missing the hour notation.

Sample Analysis Dates

Analysis of samples began on 18 Aug 2021 (date of first extraction) and concluded on 17 Dec 2021 (date of last injection). A total of 189 days transpired between the first sample collection date and the last extraction date. All study samples were analyzed within the established long-term stability of 370 days at -70°C. A total of 576 primary and 112 backup samples were analyzed in ten runs.

Sample Disposition

Sample disposition was governed by the most current version of the study protocol or QPS SOP LP-001, "Sample Chain of Custody."

Analytical Method Validation

This analysis was carried out according to the method outlined in QPS Study Number 2162-1719 entitled "Validation of a Method for the Determination of SPR206 in Human Plasma by LC-MS/MS."

Blank Matrix
Blank K2EDTA human plasma was obtained from BioIVT and used throughout the course of this study to prepare blanks, calibration standards, and QCs. Sample dilutions were performed with the same matrix, as necessary. All blank matrix was used within the expiration date provided by BioIVT.

Stock Solutions, Calibration Standards, and QCs
SPR206 stock solutions were prepared by independently weighing out reference material and quantitatively transferring to a volumetric flask with dimethyl sulfoxide. The material was dissolved and the flask was filled to volume. The concentration of the stock solution was calculated using the appropriate potency factor. All solutions were stored at approximately -20°C with precautions taken to prevent evaporative losses from the stock solutions during storage.

Calibration standards and QCs were prepared by adding the appropriate amount of analyte stock solution (within stability) and q.s. with K2EDTA human plasma or by serial dilution from a higher concentration calibration standard or QC. Calibration standard samples at eight concentration levels (50, 100, 500, 1000, 5000, 10000, 45000, and 50000 ng/mL) were prepared and stored at -70°C. Quality control samples at four concentration levels (150, 2500, 20000, and 40000 ng/mL) were prepared and stored at -70°C. Concentrations were determined based on the nominal concentrations. After preparation, the bulk standard and QC solutions were aliquoted for single use.

All stock solutions, calibration standards, and QCs were used within their respective validated stability period.

Data Acquisition and Regression Analysis
Data were acquired and processed (integrated) according to QPS SOP BA-005 using the proprietary software application Analyst (Version 1.6.2 or higher). Linear regression analysis calculations were performed with 1/x2 weighting, where x is the nominal concentration, using Watson LIMS v.7.4.1. All statistics (e.g., Mean, S.D., %CV, %RE) found in the data tables were calculated by Watson LIMS or based on the "precision as displayed" option of Microsoft Excel.

Urine Assay

Method Performance Summary

Assay Passing Rate	SPR206: Three of four runs (75%) met acceptance criteria, including ISR run.
Standard Calibration Curve	SPR206: Eight calibration standards from 100 to 50,000 ng/mL.
Standard Calibration Performance[a]	SPR206: Cumulative bias range: -3.3% to 2.8%
	SPR206: Cumulative precision: ≤9.1% CV
QC Performance[a]	SPR206: Cumulative bias range: -1.3% to 8.0%
	SPR206: Cumulative precision: ≤13.8% CV
Method Reproducibility	SPR206: Incurred sample reanalysis was performed in 15.6% of analyzed study samples, and 96.4% met the pre-specified acceptance criteria.

[a]Statistics were reported from passing runs only.

Sample Storage Conditions

Samples were received at QPS frozen on dry ice, in good condition, and upon arrival they were stored in a -70°C freezer.

Sample Collection

Urine was collected from subjects in on Day 1 at the following timepoints: prior to dosing and at 0-4, 4-8, 8-12, 12-24, 24-36 hours post-dose. Within 60 minutes of the end of the collection, each sample was treated with a Triton X-100 preservative working solution consisting of Triton-X-100:acetonitrile solution (prepared at a 1:1 volumetric ratio). The final volumetric ratio of urine:Triton X-100 preservative working solution was 100:0.2. Samples were collected from 11 Jun 2021 through 01 Dec 2021.

Sample Inventory

A total of 42 human urine primary samples of 179 total samplers were not analyzed because they thawed over two days during shipping and therefore were outside of established stability at room temperature (comments noted in QPS LIMS system). Their corresponding backup samples were analyzed instead.

Sample Analysis Dates

Analysis of samples began on 12 Nov 2021 (date of first extraction) and concluded on 14 Dec 2021 (date of last injection). A total of 186 days transpired between the first sample collection date and the last extraction date; however, no more than 178 days transpired between any individual sample collection date and its corresponding extraction date. All study samples were analyzed within the established long-term stability of 183 days at -70°C. A total of 137 primary samples and 42 backup samples were analyzed in two runs.

Sample Disposition

Sample disposition was governed by the most current version of the study protocol or QPS SOP LP-001, "Sample Chain of Custody."

Analytical Method Validation
This analysis was carried out according to the method outlined in QPS Study Number 2162-1814 entitled "Validation of a Method for the Determination of SPR206 in Human Urine by LC-MS/MS."

Blank Matrix
Blank Triton X-100 treated human urine was created by mixing human urine (collected at QPS) and Triton X-100 preservative working solution at 100:0.2 (v:v) and used throughout the course of this study to prepare blanks, calibration standards, and QCs. Sample dilutions were performed with the same matrix, as necessary. All blank matrix was used within the expiration date.

Stock Solutions, Calibration Standards, and QCs
SPR206 stock solutions were prepared by independently weighing out reference material and quantitatively transferring to a volumetric flask with dimethyl sulfoxide. The material was dissolved and the flask was filled to volume. The concentration of the stock solution was calculated using the appropriate potency factor. All solutions were stored at approximately -20°C with precautions taken to prevent evaporative losses from the stock solutions during storage.

Calibration standards and QCs were prepared by adding the appropriate amount of analyte stock solution (within stability) and q.s. with Triton X-100 treated human urine or by serial dilution from a higher concentration calibration standard or QC. Calibration standard samples at eight concentration levels (100, 200, 500, 1500, 5000, 15000, 45000, and 50000 ng/mL) were prepared and stored at -70°C. Quality control samples at four concentration levels (300, 2500, 20000, and 40000 ng/mL) were prepared and stored at -70°C. Concentrations were determined based on the nominal concentrations. After preparation, the bulk standard and QC solutions were aliquoted for single use. All stock solutions, calibration standards, and QCs were used within their respective validated stability period.

Data Acquisition and Regression Analysis
Data were acquired and processed (integrated) according to QPS SOP BA-005 using the proprietary software application Analyst (Version 1.6.1 or higher). Linear regression analysis calculations were performed with 1/x2 weighting, where x is the nominal concentration, using Watson LIMS v.7.4.1. All statistics (e.g., Mean, S.D., %CV, %RE) found in the data tables were calculated by Watson LIMS or based on the "precision as displayed" option of Microsoft Excel.

Hemodialysis Procedure
Blood flow rate and dialysis flow rate are for the 1st and 2nd hemodialysis procedure
Fresenius 4008B; blood flow rate 250, 250; dialysis flow rate 500, 500
Fresenius 4008B; blood flow rate 230, 250; dialysis flow rate 500, 500
Fresenius 4008B; blood flow rate 220, 250; dialysis flow rate 500, 500
Baxter Revaclear 400; blood flow rate 300, 300, 206; dialysis flow rate 500, 500
Gambro Revaclear 400; blood flow rate 300, 350; dialysis flow rate 500, 500
Baxter Revaclear 400; blood flow rate 300, 300; dialysis flow rate 500, 500
Baxter Revaclear 400; blood flow rate 300; 330; dialysis flow rate 500, 550

Device	Fluid	Visit Day	Sample Number	Mean	Standard Deviation	Min	Max
Fresenius 4008B	Blood	1	3	233.3	15.28	220.0	250.0
		5	3	250.0	0.00	250.0	250.0
	Dialysis Fluid	1	3	500.0	0.00	500.0	500.0
		5	3	500.0	0.00	500.0	500.0
Baxter Gambro Revaclear 400	Blood	1	4	300.0	0.00	300.0	300.0
		5	4	296.5	63.74	206.0	350.0
	Dialysis Fluid	1	4	500.0	0.00	500.0	500.0
		5	4	512.5	25.00	500.0	550.0

Min, minimum; Max, maximum.
